# Age- and Sex-Stratified Normogram Study of Corpus Callosum Dimensions in South Indian Individuals

**DOI:** 10.7759/cureus.72822

**Published:** 2024-11-01

**Authors:** Siva Sidhanth Y, Harini Bopaiah, Anil Kumar Sakalecha, Anees Dudekula, Rishi Prajwal H L, Priyanka Punuru

**Affiliations:** 1 Radiodiagnosis, Sri Devaraj Urs Medical College, Kolar, IND; 2 Radiology, Sri Devaraj Urs Medical College, Kolar, IND

**Keywords:** atrophy, brain, corpus callosum, imaging, magnetic resonance imaging, splenium

## Abstract

Background

The corpus callosum (CC) is an important structure of the brain consisting of various parts. The morphometric examination of physiological fluctuations in CC morphology is critical, as these variations may complicate the diagnosis and management of the individual. Therefore, it is important to assess and determine the range of such parameters.

Materials and methods

An observational, cross-sectional prospective hospital study was carried out over a period of one year, wherein 110 subjects attending our institution were assessed for the mean and range of different parts of the CC across equally distributed males and females, with the help of standardized parameters using MRI.

Results

The mean anteroposterior (AP) diameter of CC and the mean thickness of the genu, body, isthmus, and splenium were found to be 65.56±8.75 mm, 7.89±2.54 mm, 5.91±1.77 mm, 4.85±1.57 mm, and 8.57±1.92 mm, respectively. The AP diameter shows the greatest variability, with mean values increasing with age, whereas all other parameters such as the thickness of the genu, body, isthmus, and splenium were found to decrease with age. An assessment of these parameters between either gender revealed higher values among males in comparison to that of females, which was found to be statistically significant (p<0.05) across all parameters among the male and female genders.

Conclusion

The analysis of CC dimensions shows significant variability affected by age and gender. This study offers crucial baseline data on CC morphology, highlighting the important differences between males and females. Recognizing these normal anatomical variations can significantly improve clinical assessments of individuals with potential neurological issues. Further research is needed to investigate the clinical implications of these morphometric differences across different neurological contexts, which may lead to enhanced diagnostic and management approaches for affected individuals.

## Introduction

The greatest group of commissural fibers in the brain is the corpus callosum (CC) [1]. The rostrum, genu, body, isthmus, and splenium are the five components that make up the CC [1-4]. The genu connects the lateral and medial frontal lobes of both sides [1-4]. The body connects the sections of each hemisphere cortex, which interact with the corona radiata. The isthmus links the pre- and postcentral gyri, as well as the auditory cortex for each hemisphere, to their respective counterparts [1-4]. The posteriormost structure is the splenium which predominantly connects temporal, posterior parietal, and occipital cortices [1-4].

Morphometric analysis of physiological changes in CC architecture is crucial. These variations may complicate the diagnosis and treatment of neuropsychiatric illnesses, dysmyelinating and demyelinating disorders, prenatal and neonatal trauma, and hypoxic injury [5].

Neurologists and anatomists have debated and investigated the structure of CC and the relationship between the changes in its dimensions with intelligence and comprehension [6]. According to research, it has a role in complex brain operations like learning, memory, reasoning, three-dimensional vision, executive functions, and behavioral patterns [7].

The thickness of the CC changes in a number of conditions, including white matter diseases like multiple sclerosis, dementia, tumors, cerebrovascular diseases, acquired immunodeficiency syndrome (AIDS), chemotherapy, handedness, autism, etc. As a result, the thickness of different CC parts provides information about the severity of the white matter abnormality [7,8].

Several previous studies found that CC morphology varied with age and gender and in certain illnesses such as bipolar disorders, vascular dementia, Alzheimer's disease, Williams syndrome, and other disease processes [1,3,8].

MRI is one of the most efficacious diagnostic modalities, as it provides us with all vital information, which can be unclear or deficient in other conventional diagnostic modalities [9]. In the case of CC, it has been put forward that the mid-sagittal T1-weighted MR sequences are appropriate for seeing and measuring the thickness of different portions of the CC [9].

Over the years, few studies have reported the mean values for CC subregions [1]. Our study was designed to assess age- and gender-related changes in CC subregions across the South Indian population with the use of MRI while comparing the same across either gender.

## Materials and methods

An observational, cross-sectional prospective hospital study was carried out over a period of one year (2023-2024), wherein 110 subjects attending the Department of Radiodiagnosis at R. L. Jalappa Hospital in Sri Devaraj Urs Medical College formed a part of our study.

Prior to the start of the study, informed consent was taken from all the patients who were part of our study. We also obtained ethical clearance from the Central Ethics Committee of Sri Devaraj Urs Academy of Higher Education and Research (approval number: SDUAHER/KLR/R&D/CEC/S/PG/50/2024-25) prior to the start of the study with advantageous propositions being included within the design of our study.

The said committee has unanimously approved the PG project titled "Age- and Sex-Stratified Normogram Study of Corpus Callosum Dimensions in South Indian Individuals" and granted permission to investigators to carry out the research work.

Inclusion criteria

The study included patients aged 7-77 years who were referred for MRI evaluation due to various conditions, such as headaches, seizures, balance disorders, trigeminal neuralgia, and visual abnormalities, all of which resulted in normal MRI findings. Additionally, older patients who underwent MRI for various reasons and exhibited mild periventricular ischemic changes related to aging were also included.

Exclusion criteria

Patients were excluded if they had a history of trauma, hydrocephalus, inborn errors of metabolism, space-occupying lesions, demyelinating or dysmyelinating disorders, or infarcts causing volume loss and other pathological processes.

Methodology

Patients who met the inclusion criteria underwent brain MRI on a 1.5 T, 18-channel MR scanner (Siemens Magnetom Avanto®, Erlangen, Germany) after being informed and providing consent. 

**Figure 1:**
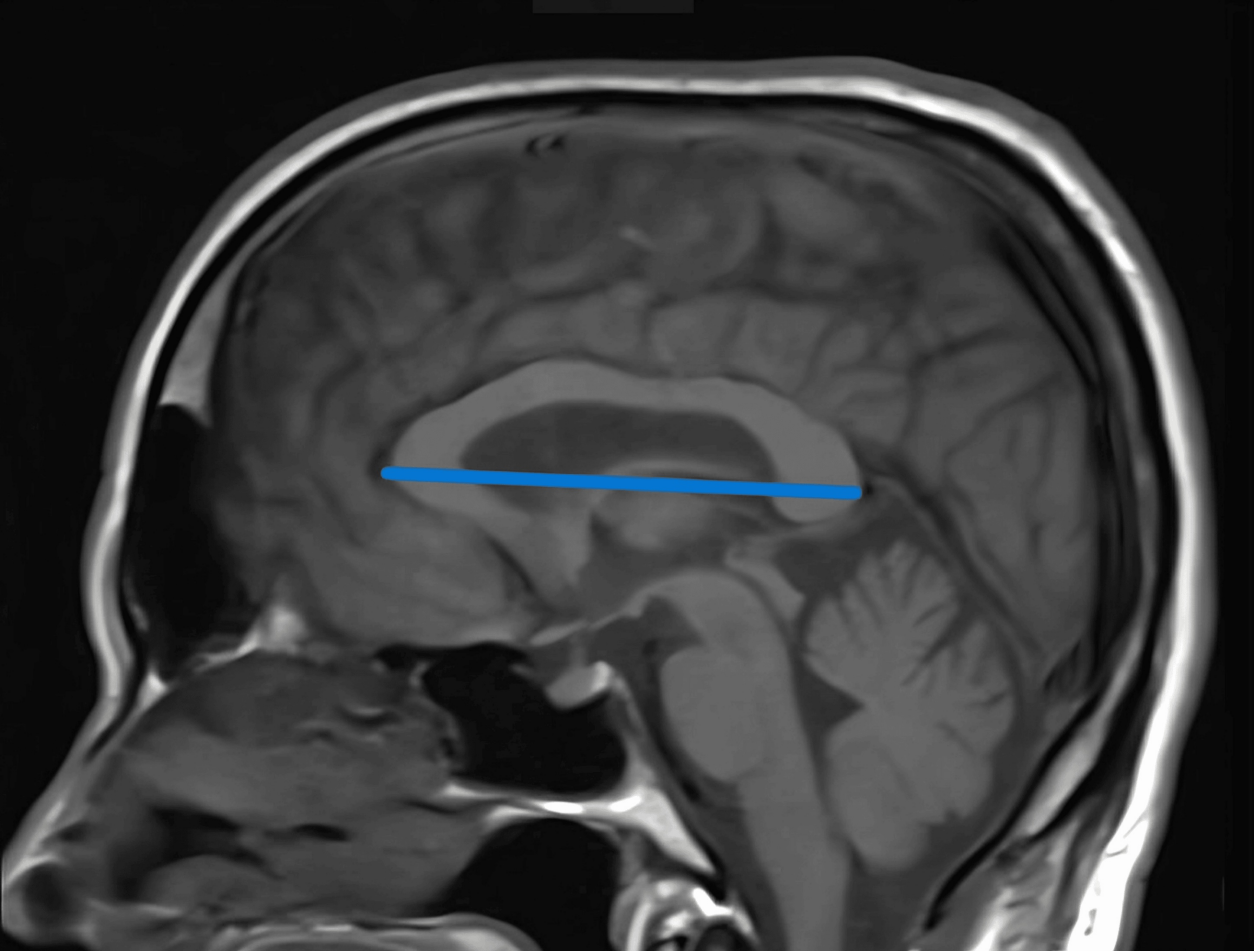
Mid-sagittal T1-weighted MR image showing the measurement of the anteroposterior diameter of the corpus callosum: distance between the anterior aspect of the genu and the posterior aspect of the splenium

**Figure 2:**
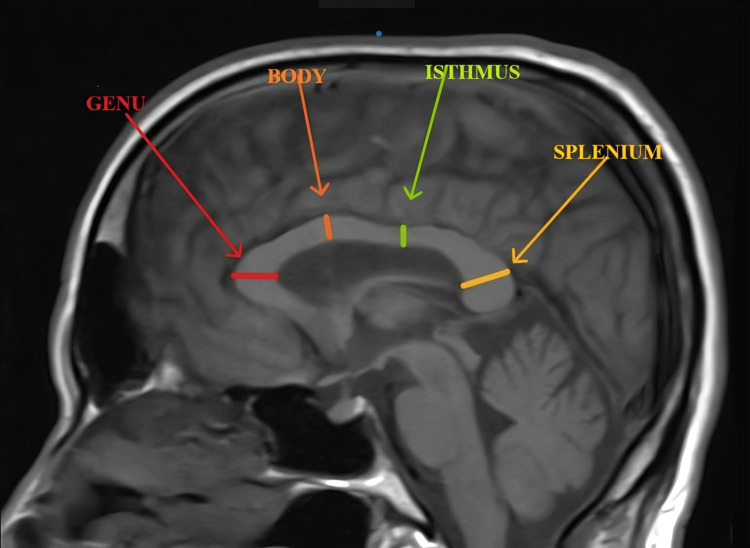
Mid-sagittal T1-weighted image showing the measurement of the maximum thickness of various parts of the corpus callosum

The entire measurement was taken in the mid-sagittal plane, by identifying the midpoints of the posterior and anterior commissures on sagittal T1-weighted MR images. The CC's anteroposterior (AP) diameter and the maximum thickness of the genu, splenium, isthmus, and rostrum were measured as described in Figure 1 and Figure 2 below.

Statistical analysis

To facilitate analysis, the collected data was stratified by age and gender. The data was imported into Microsoft Excel (Microsoft Corporation, Redmond, Washington, United States) and analyzed using IBM SPSS Statistics for Windows, Version 22.0 (Released 2013; IBM Corp., Armonk, New York, United States). The patient's sociodemographic and clinical parameters were summarized using mean (SD) and range for continuous variables. Student's t-test was used to compare continuous variables (AP diameter of the CC and thickness of the genu, body, isthmus, and splenium) between males and females. P-values less than 0.05 will be considered statistically significant.

## Results

Mean and range of various parameters of CC

Table 1 presents the dimensions of the CC, indicating that the AP diameter exhibits the widest range and mean value. The maximum thickness of the genu and splenium have the largest mean values among the segments of CC, while the body and isthmus show smaller dimensions. Overall, these measurements provide valuable normative data for assessing CC anatomy.

**Table 1:** Mean and range of various parameters of the corpus callosum AP: anteroposterior

Parameters	Range	Mean±SD
AP diameter (mm)	53.46-84.17	65.56±8.75
Genu (mm)	3.9-15.32	7.89±2.54
Body (mm)	2.32-9.67	5.91±1.77
Isthmus (mm)	2.03-7.23	4.85±1.57
Splenium (mm)	5.5-13.45	8.57±1.92

Various parameters of CC across different age groups

Table 2 displays the mean values of various parameters of the CC across different age groups. Notably, the AP diameter increases with age, with the highest mean observed in the 54-77-year age group. In contrast, the genu shows an increase in mean value from the 7-30 to the 31-53 but decreases in the 54-77-year age group. The body and isthmus parameters reveal a slight decline in mean values with advancing age, while the splenium exhibits a significant decrease from the younger to older age groups. 

**Table 2:** Mean value of various parameters of the corpus callosum across different age groups AP: anteroposterior

Parameters	Years	Mean (mm)
AP diameter	7-30	62.40±10.92
	31-53	64.12±8.14
	54-77	70.16±8.75
Genu	7-30	6.88±3.24
	31-53	9.27±2.55
	54-77	7.52±2.34
Body	7-30	6.18±1.98
	31-53	5.95±1.65
	54-77	5.60±1.71
Isthmus	7-30	4.98±1.89
	31-53	4.81±1.48
	54-77	4.76±1.63
Splenium	7-30	10.32±2.24
	31-53	8.05±1.68
	54-77	7.34±1.86

Gender-wise comparison of various parameters of CC

Table 3 highlights significant differences in the dimensions of the CC between genders across multiple parameters. Males consistently show larger mean values in all measured dimensions: the AP diameter (69.27 mm vs. 61.86 mm), genu (8.87 mm vs. 6.92 mm), body (6.42 mm vs. 5.40 mm), isthmus (6.12 mm vs. 3.58 mm), and splenium (9.61 mm vs. 7.54 mm).

**Table 3:** Mean dimensions of the corpus callosum across genders * indicates significant p-value AP: anteroposterior

Parameters	Gender	Mean±SD (mm)	P-value
AP diameter	Males	69.27±10.11	<0.00001*
	Females	61.86±4.91	
Genu	Males	8.87±2.65	<0.00001*
	Females	6.92±6.92	
Body	Males	6.42±1.83	<0.001*
	Females	5.40±1.55	
Isthmus	Males	6.12±0.96	<0.00001*
	Females	3.58±0.88	
Splenium	Males	9.61±1.80	<0.00001*
	Females	7.54±1.42	

The statistically significant p-values (<0.00001 for the AP diameter, genu, isthmus, and splenium; <0.001 for the body) indicate strong evidence that these differences are not due to chance. These findings suggest that male individuals typically have larger CC dimensions than females, emphasizing the need to consider gender differences in neuroanatomical studies.

## Discussion

The CC is the primary interhemispheric white matter pathway, with the potential to be impacted by both physiological and pathologic processes occurring in both cortical and subcortical regions [1,4,10].

According to Krishna et al.'s research, the thickness and AP length of the CC change with age. AP length increased with age, and it was larger in people aged over 60 [1]. The thickness of several regions of the CC was higher in young adults than in children, adolescents, and the elderly [1].

The mean AP diameter of CC across our study subjects was found to be 65.56 mm with a range of 53.46-84.17 mm, wherein the mean AP diameter was found to increase with age. The mean AP diameter among males and females was noted to be 69.27 mm and 61.86 mm, which was found to be statistically significant.

In a similar study by Pasricha et al., they discovered the average length of CC to be 73.53±4.28 mm with a mean age of 49.05±19.7 years, ranging from 20 to 80 years [5].

As per Krishna et al. [1], the mean AP diameter of the CC increased gradually with age, reaching 72.75 mm in the age range of 61-70 years. However, in a study of 100 patients by Suganthy et al., the length of the CC increased significantly with age in both males and females, with a mean value of 72.6 mm in the over-60 age group [10]. Guz et al. [11] found that this parameter gradually increases with age, reaching a mean value of 68.93±4.5 mm in the 61-70-year age group.

Similarly, in a study by Allouh et al., children (2-10 years) had considerably shorter AP lengths of the CC compared to younger (20-45 years) and older adults (55-80 years), with mean values of 60.5±5.3 mm, 68.4±4.0 mm, and 69.5±4.1 mm [2].

On further assessment, we found the mean genu diameter to be 7.89 mm with a range of 3.9-15.32 mm, with the mean genu diameter decreasing with age. The mean genu diameter among males and females was noted to be 8.87 mm and 6.92 mm, which was found to be statistically significant.

However, Pasricha et al. [5] found the genu to be 5.16 mm. Further, in terms of the age correlation, Krishna et al. [1] reported the mean thickness values of the genu showed a gradual increase followed by a plateau at the age of 20-40 years and thereby decreased in thickness, which was almost similar to that observed in our study.

The mean body diameter among our subjects was found to be 5.91 mm with a range of 2.32-9.67 mm, with the mean body diameter decreasing with age. The mean body diameter among males and females was noted to be 6.42 mm and 5.40 mm, which was found to be statistically significant.

According to Krishna et al. [1], the largest mean thickness of the body was observed in the age group of 11-20 years, followed by a progressive decline in thickness with increasing age, with a lowest mean value of 4.99 mm in the age group of 61-70 years, which was also agreed upon by Jain et al. who discovered a substantial decrease in breadth, particularly of the genu and body, with increasing age [3].

This was also in agreement with that of Gupta et al. [12], who observed that the diameter of the CC body was greater in the young age group of 20-40 years than in the older age group of >40 years. Furthermore, the width of the genu and trunk reduced with age in males [10].

Other parameters like the mean isthmus diameter were found to be 4.85 mm with a range of 2.03-7.23 mm, wherein the mean isthmus diameter was found to be decreasing with age. The mean isthmus diameter among males and females was noted to be 6.12 mm and 3.58 mm, which was found to be statistically significant.

A similar plateau was reported by Krishna et al. [1], who had the mean thickness of the isthmus gradually increased from 1-10 years to 30 years and then declined with age. Tanaka-Arakawa et al. [13] discovered that the average thickness of the isthmus was greater in the adult age group of 18-25 years.

Also, the mean splenium thickness was found to be 8.57 mm with a range of 5.5-13.45 mm, wherein the mean splenium thickness was found to be commensurately decreasing with age. The mean splenium thickness among males and females was noted to be 9.61 mm and 7.54 mm, which was found to be statistically significant.

Almost similar results were reported by Pasricha et al. [6], wherein they found the mean splenium thickness to be 9.94 mm. However, other studies reported higher values in comparison to that observed in our study.

Ajare et al. [14] found the mean splenium thickness to be 11.01 mm, while Junle et al. [15] reported the mean splenium thickness to be 11.53 mm. Also, Allouh et al. found it to be 16.65 mm which was the highest across the studies we assessed [2].

Most of our study results were in agreement with that of the findings from other populations, thereby indicating a decrease in thickness of the body, rostrum, and splenium with age, which is linked to the generalized degeneration of cortical neurons or atrophy of white matter with advancing age [16-19].

Recognizing age-related changes in the CC can aid in differentiating between normal aging and pathological conditions, such as multiple sclerosis or Alzheimer's disease. MRI and other imaging techniques can be employed to monitor these changes, leading to earlier diagnosis and intervention.

Providing patients with information about how aging affects brain structure can empower them to take proactive steps in maintaining cognitive health through lifestyle changes and cognitive training.

Limitations

Even though our study was designed to assess the normative measurements of CC among the South Indian population, with a small, limited, and single-centric sample, it is difficult to exactly determine a precise value; hence, we recommend a larger, multicentric population of the geographic area to make the results more reliable.

## Conclusions

This study provides valuable normative data on the dimensions of the CC across different age groups and genders within a South Indian population. Our findings reveal significant variability affected by age and gender, with males generally exhibiting larger dimensions than females. The AP diameter shows the greatest variability, with mean values increasing with age, whereas all other parameters such as the thickness of the genu, body, isthmus, and splenium were found to decrease with age.

Recognizing these normal anatomical variations can significantly improve clinical assessments of individuals with potential neurological issues, which can help distinguish between normal aging processes and pathological conditions. Further research with larger, multi-centric cohorts is recommended to validate these findings and enhance their applicability in clinical contexts, particularly in the assessment and early diagnosis of neurodegenerative diseases.
